# Physicobiochemical Characteristics and Chondrogenic Differentiation of Bone Marrow Mesenchymal Stem Cells (hBM-MSCs) in Biodegradable Porous Sponge Bovine Cartilage Scaffold

**DOI:** 10.1155/2019/8356872

**Published:** 2019-01-20

**Authors:** Dwikora Novembri Utomo, Ferdiansyah Mahyudin, Teddy Heri Wardhana, Purwati Purwati, Febrian Brahmana, Agrippina Waya Rahmaning Gusti

**Affiliations:** ^1^Department of Orthopaedics and Traumatology, Faculty of Medicine, Universitas Airlangga/Dr. Soetomo General Hospital, Surabaya, Indonesia; ^2^Stem Cell Research and Development Center, Universitas Airlangga, Surabaya, Indonesia; ^3^Biomedical Engineering Program, Postgraduate School, Universitas Airlangga, Surabaya, Indonesia

## Abstract

Tissue engineering had been believed to overcome the limitation of cartilage lesions treatment. Nowadays the studies focus on using mesenchymal stem cells in scaffold. A biodegradable porous sponge bovine cartilage scaffold is expected to have the physicobiochemical characterization to promote chondrogenic differentiation of hBM-MSCs. Scaffold from bovine cartilage was printed in 5 mm diameter sponge, categorized into nondecellularized (SBCS) and decellularized (DSBCS). Physical characteristics (pore diameter and interconnectivity) were done using a Scanning Electron Microscope (SEM). Biodegradability assessment used Phosphate Buffered Saline in 15, 30, 60 minutes, 6, 24, 48, 72 hours, and 1, 2 weeks. The swelling ratios were counted in 5, 10, 15, 30, 60, and 360 minutes. Biochemical characteristics were obtained by enzyme-linked immunosorbent assay for type II collagen, aggrecan, and Transforming Growth Factors-*β* (TGF-*β*). Data were statistically compared. hBM-MSCs were seeded on both scaffolds. Histological examination used hematoxylin-eosin taken at the 2nd and 4th weeks after seeding. There was no significant difference (p=0.473; p=0.142) on mean porosity 90.07 ± 4.64% vs. 88.93 ± 4.18% and pore diameter 111.83 ± 14.23 *μ*m vs. 105.29 ± 11.14 *μ*m assessment between SBCS and DSBCS groups. Scaffolds from both groups showed pore interconnectivity. DSBCS group had faster biodegradability. SBCS group sweals better. SBCS group contains type II collagen, aggrecan, and TGF-*β* with mean values 380.78 ± 18.63 ng/ml, 30.71 ± 4.50 ng/ml, and 130.12 ± 7.73 ng/ml, respectively, while DSBCS contained type II collagen, aggrecan, and TGF-*β* with mean values 64.83 ± 13.54 ng/ml, 8.41 ± 2.38 ng/ml, and 16.39 ± 4.49 ng/ml, respectively. The results were statistically different (p<0.001). Chondrocytes were found within scaffold on the 2nd and 4th weeks. Physicobiochemical characteristic of biodegradable sponge bovine cartilage scaffold promotes chondrogenic differentiation of hBM-MSCs.

## 1. Introduction

Articular cartilage is a special connective tissue, lining the diarthrodial joint to protect the subchondral bone, providing a smooth, oily surface and facilitating the transmission of loads with a low coefficient of friction in the joints [1, 2].

Trauma and degenerative processes may damage articular cartilage, resulting in joint pain which decreases the quality of life and raises the possibility of long-term complications such as osteoarthritis [3, 4]. Articular cartilage has a very limited intrinsic healing capability due to low cellularity, being avascular and aneural properties [5, 6]. Osteochondral damage often results in secondary fibrocartilage tissue due to an inflammatory response. Fibrocartilage tissue has worse biomechanical properties than hyaline cartilage tissue, which can lead to early degradation and fragmentation of articular cartilage [7].

Management for articular cartilage damage is still a challenge for orthopedic surgeons. Current therapeutic options are very diverse, ranging from nonsurgical therapy which includes drugs, such as nonsteroidal anti-inflammatory drugs (NSAIDs), corticosteroid injections, or viscosupplementation, then followed by lifestyle modification, weight loss, bracing, and physiotherapy. Surgical therapy option includes arthroscopic lavage and debridement, marrow tapping techniques, abrasion arthroplasty, subchondral drilling, microfracture, osteochondral allo/autografting techniques, autologous cell-based techniques, conventional autologous chondrocyte implantation (ACI), matrix-induced ACI (MACI), and growth factors injection to genetic-based therapy. There is no evidence of an improvement in the quality of structure of articular cartilage through nonsurgical therapy. Subchondral drilling can cause thermal necrosis, on the other hand, microfracture techniques provide good results, though only limited to patients older than 40 years. Autologous chondrocyte implantation has the disadvantages of multiple surgical procedures, wider surgical wounds, donor site morbidity, and periosteum flap complications such as cell leak, peripheral hypertrophy, and calcification problems that cause a clinical condition of “catching” knee [8].

All of the above therapeutic options generally produce fibrocartilage tissue, which is unable to produce sufficient hyaline cartilage tissue. Fibrocartilage tissue is susceptible to damage when receiving normal knee compression loads [8]. One of the current therapeutic strategies is the tissue engineering approach to repair and regenerate cartilage with the same biomechanical characteristics, biological composition, and structure resembling the original articular cartilage. Tissue engineering triad consists of three components: cells, signals, and scaffolds.

Scaffold acts as a three-dimensional template to cover cellular defects, distribution, proliferation, and differentiation. Scaffold must be biodegradable, nontoxic, able to integrate with host tissue, maintain life, cell phenotype during implantation in vitro and in vivo. Scaffold should have interconnective pores that are highly porous to support cell growth, nutrients transport, and remove metabolic waste products [9, 10]. Sponge porous structure is expected to absorb nutrients for stem cells culture in vitro and joint fluid on in vivo applications.

Organic materials are preferred because they have better biocompatibility and biodegradability than synthetic materials [11]. Bovine cartilage scaffold sponge was a byproduct, without economic value, and has been previously discarded. This biomaterial is cheap and easy to acquire. Bovine cartilage scaffold will not damage the stem cell [12].

There is no characteristic study regarding physical characteristics, biochemistry, biodegradation, and the ability of sponge bovine cartilage scaffold to facilitate chondrogenic differentiation of hBM-MSCs in an attempt to prove that sponge bovine cartilage scaffold apart is an ideal scaffold, which provides biochemical signaling, ability to facilitate chondrogenic differentiation of hBM-MSCs. The aim of this study is to prove that sponge bovine cartilage scaffold in cartilage tissue engineering may treat articular cartilage defect.

## 2. Materials and Methods

The study was an in vitro laboratory study on sponge bovine cartilage scaffold with posttest only design. Scaffold was divided into two groups, a group without decellularization process (SBCS) and a group with decellularization process (DSBCS). This study compared the physical, biochemical characteristics, biodegradability, water uptake ability, and ability to facilitate chondrogenic differentiation of hBM-MSCs between SBCS group and DSBCS group. The physical properties include porosity, pore diameter size, and interconnectivity while biochemical characteristics include type II collagen, aggrecan, and TGF-*β*.

### 2.1. Synthesis of Sponge Bovine Cartilage Scaffold

Sponge bovine cartilage scaffold is biomaterial taken from bovine's femoral head and femoral condyle of at least 24 months old provided certified abattoirs in accordance with the study inclusion criteria. Cartilage is separated from the bone, then it was washed using 0.9% NaCl solution or distilled water and later processed into powder by grinding. Cartilage has been processed in the form of powder then filtered by test sieve with frame size of 150 *μ*m-355 *μ*m. Cartilage granules, with size of 150 *μ*m-355 *μ*m, mixed with distilled water or 0.9% NaCl in a ratio of cartilage powder : distilled water or 0.9% NaCl was 1:1. The mixture was placed into a 5-cm diameter of sponge mold first.

**Figure 1:**
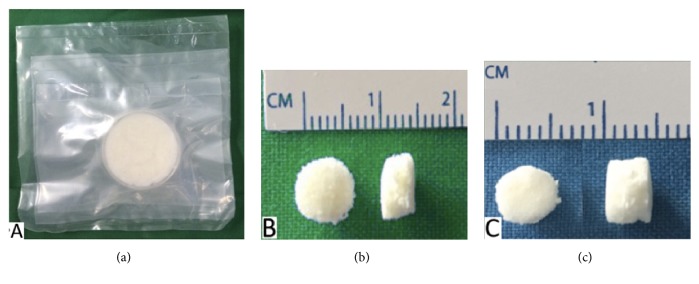
(a) Sponge bovine cartilage scaffold 5 cm in diameter, (b) sponge bovine cartilage scaffold in SBCS group, 5 mm in diameter, and (c) sponge scaffold in DSBCS group, 5 mm in diameter.

The mold containing a mixture of cartilage powder and distilled water was then frozen in a freezer with a temperature of −80°C for at least 24 hours with deep frozen technique. After being frozen, the mixture was dried with sublimation techniques of freeze-dried method using freeze-dryer machine. After it was dried, sponge cartilage bovine is reprinted with a three-dimensional mold, 5 mm in diameter. DSBCS group was decellularized physically (freeze-thaw) and chemically by sodium dodecyl sulfate (SDS) 5% for 72 hours. The result was shown in Figure 1.

### 2.2. Physical Characteristics Measurement Using Scanning Electron Microscope (SEM)

Physical characteristics were analyzed through Scanning Electron Microscope examination (Inspect S50) to evaluate pore diameter, interconnectivity, morphology, and topography of the sample surface.

### 2.3. Porosity Measurement

Porosity is calculated using the following formula:

(1) $$\begin{array}{c} Porosity=\frac{\left(W_{2}-W_{1}\right)}{\left(W_{2}-W_{3}\right)}\times 100\% \end{array}$$

 
W_1_: dry weight of the scaffold
 
W_2_: wet weight of scaffold in PBS liquid
 
W_3_: the weight of the scaffold in PBS subtracting buoyancy liquid from W_1_

### 2.4. Biodegradability Test

Sponge bovine cartilage scaffold was immersed in 20 ml PBS (pH 7.4) with the temperature of 37°C. Initial dry weight (W_i_) was measured then soaked for 15, 30, 60 minutes, 6, 24, 48, 72 hours, and 1 week and 2 weeks. Scaffold was dried on a filter paper, in a room temperature of 37°C for 24 hours then (W_f_) was measured. Weight loss was calculated by the following formula:

(2) $$\begin{array}{c} \text{Weight loss }\left(\;\%\;\right)=\frac{\left(W_{i}-W_{f}\right)}{W_{i}}\times 100 \end{array}$$

### 2.5. Water Uptake Test (Swelling Ratio)

Scaffold dry weight (W_i_) was soaked in PBS pH 7.4 at room temperature of 37°C. Scaffold wet weight (W_w_) was at 10, 15, 30, 60 minutes and 6 hours. The swelling ratio was calculated by the following formula:

(3) $$\begin{array}{c} \text{Water uptake }\left(\;\%\;\right)=\frac{\left(W_{w}-W_{i}\right)}{W_{i}}\times 100 \end{array}$$

### 2.6. Biochemical Testing Using Enzyme-Linked Immunosorbent Assay (ELISA) Reader

Biochemical characteristic was obtained using enzyme-linked immunosorbent assay (ELISA) for type II collagen, aggrecan, and TGF-*β* level. Scaffold must be pounded first into powder form. Then it was dissolved, centrifuged, and processed to obtain the supernatant and later submitted into the ELISA Reader.

### 2.7. Histological Examination

Human bone marrow mesenchymal stem cells (hBM-MSCs) were processed to the fifth passage. CD45, CD105, CD73, and CD90 markers were analyzed immunohistochemically to confirm cell characteristic. The number of cells that were seeded to each scaffold was 1.55 × 10^6^.

Hematoxylin-eosin staining was performed. Examination with light microscope by an observer on cultured scaffolds with hBM-MSCs was carried out in 2 and 4 weeks. Presence of chondrocytes within the SBCS group and DSBCS group has been documented and calculated.

## 3. Results

### 3.1. Physical Characteristics of Sponge Cartilage Bovine Scaffold

**Figure 2:**
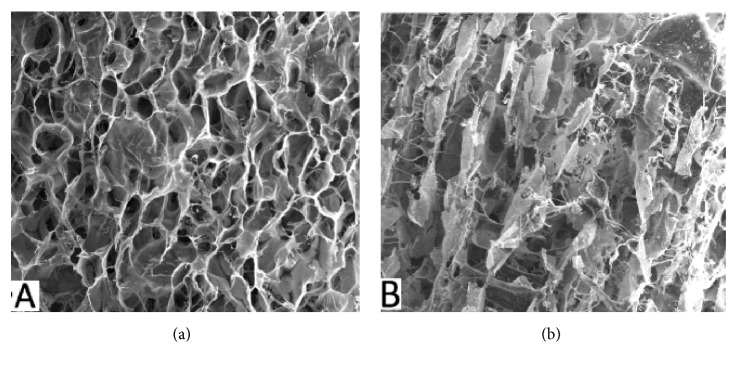
Cross-sectional examination of scaffold using SEM. (a) SBCS group and (b) DSBCS group.

Cross-sectional examination by Scanning Electron Microscopy (SEM) showed that the shape and size of porous in the SBCS group were more homogeneous compared to the DSBCS group which can be seen from Figure 2.

**Figure 3:**
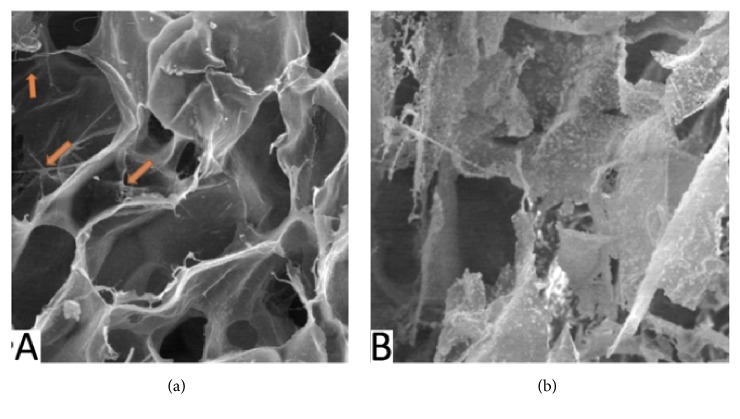
Cellular component in (a) SBCS group and (b) DSBCS group. Red arrow showed a cellular component of SBCS group.

Figure 3 showed the presence of cellular component in the SBCS group, whereas in the DSBCS group was absent.

**Figure 4:**
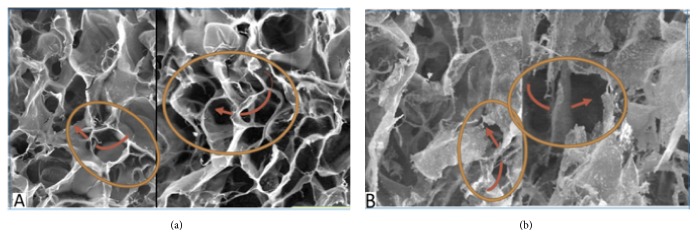
Pore interconnectivity in (a) SBCS group and (b) DSBCS group. Red arrow showed interconnectivity pore in area consisting of some pores within orange circle.

Pore interconnectivity as shown in Figure 4 was observed in both scaffold groups. Pore size diameter in the SBCS group was 34.8 *μ*m to 372.5 *μ*m and in the DSBCS group was 14.7 *μ*m to 332.7 *μ*m. The average pore diameter in SBCS group was 111.83 ± 14.23 *μ*m and in DSBCS group was 105.29 ± 11.14 *μ*m. The difference was not statistically significant (p=0.142) as shown in Table 2.

Table 1 showed pore size distribution based on diameter; most of pores on both groups had diameter range between 50 *μ*m–150 *μ*m. Porosity 90.07 ± 4.64% was found in SBCS group and 88.93 ± 4.18% in DSBCS group, but there was not statistically significant difference between the two groups (p=0.473) as shown in Table 2.

### 3.2. Biodegradability of Sponge Bovine Cartilage Scaffold

Biodegradability as mass was reduced in PBS solution (pH 7.4) in room temperature of 37°C calculated at 15, 30, 60 minutes, 6, 24, 48, 72 hours, 1 week and 2 weeks on both scaffold groups.

**Figure 5:**
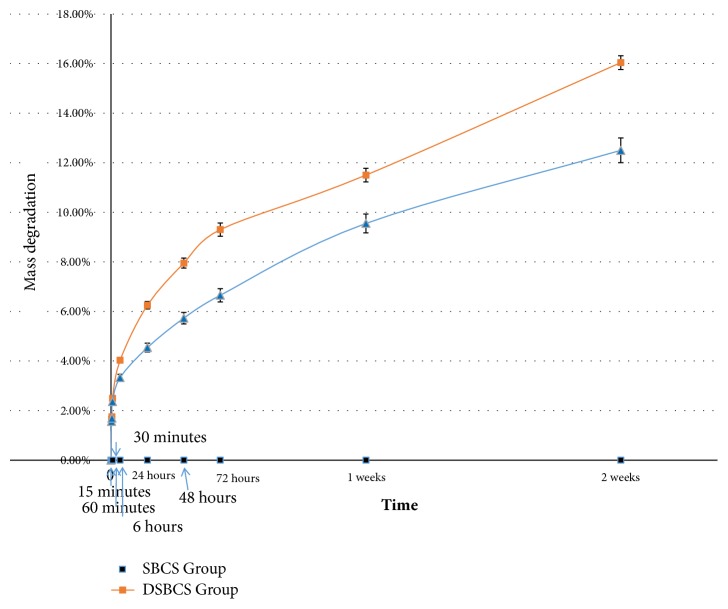
Plot of biodegradation of both sponge bovine cartilage scaffold groups at 15, 30, 60 minutes, 6, 24, 48, 72 hours, 1 and 2 weeks. The error bars represent the standard deviation of measurements for 9 points of time in 6 separate sample runs (n = 54) in each scaffold group.

Biodegradation rate in SBCS and DSBCS groups at first 6 hours was relatively the same, but after the first 24 hours, DSBCS group was degraded faster as shown in Figure 5. General linear model test showed the difference between the two groups was statistically significant (p<0.001).

### 3.3. Water Uptake Ability (Swelling Ratio)

**Figure 6:**
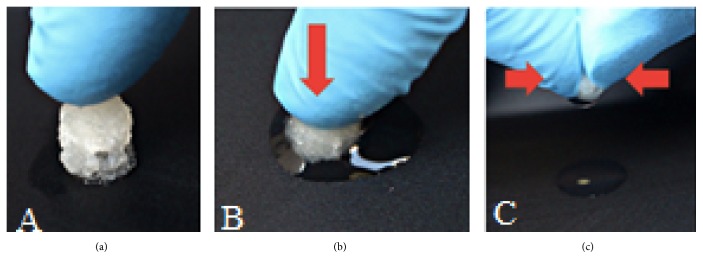
Water uptake ability and retention of SBCS group. (a) Shortly after being removed from the liquid bath, (b) when it was pressed, the liquid came out from the scaffold, (c) when squeezed and pressed harder, the residual liquid still coming out from the scaffold.

**Figure 7:**
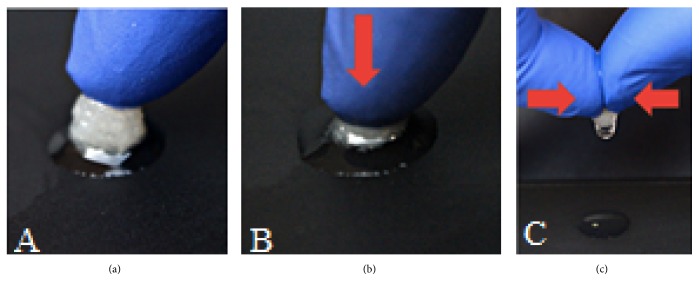
Water uptake ability and retention of the DSBCS group. (a) Shortly after being removed from the liquid bath, (b) when it was pressed, the liquid gushed out of the scaffold, (c) when squeezed pressed harder, the residual liquid still coming out from the scaffold.

Sponge cartilage bovine scaffold had good water absorption and retention. Figures 6 and 7 showed the appearance, water absorption, and retention of SBCS and DSBCS groups. Scaffold can be formed into different shapes with a mold, which satisfies the requirement for clinical application.

**Figure 8:**
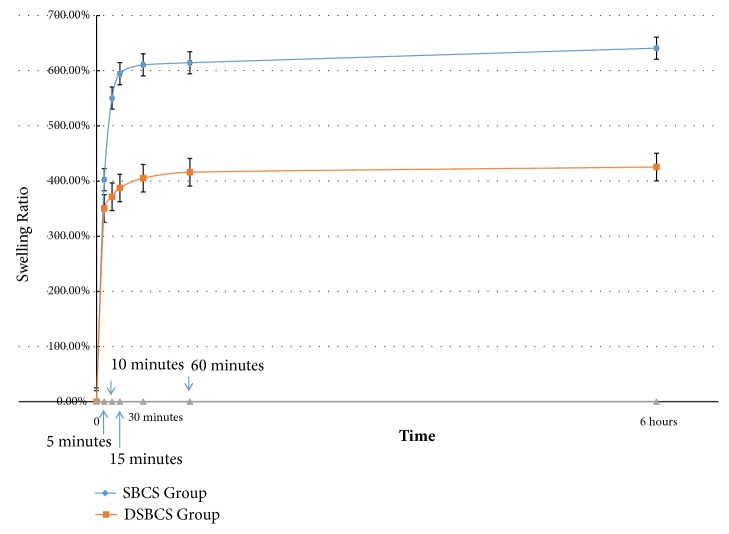
Plot of swelling ratio assessment in 6 hours. The error bars represent the standard deviation of measurements for 6 points of time in 5 separate sample runs (n = 30) in each scaffold group.

Swelling ratio measurement was done at 5, 10, 15, 30, 60 minutes and 6 hours. At first fifteen minutes, water absorption was higher in the SBCS group 594.66 ± 5.08% than DSBCS group 387.50 ± 2.35%. The SBCS group was able to absorb water more than the DSBCS group. The results of swelling ratio assessment for 6 hours period were illustrated in Figure 8. General linear model test showed the difference between the two groups was statistically significant (p<0.001).

### 3.4. Biochemical Test Results of Sponge Bovine Cartilage Scaffold

Type II collagen levels obtained in the SBCS group were 380.78 ± 18.63 ng/ml and in the DSBCS group were 64.83 ± 13.54 ng/ml. The mean level of type II collagen was higher in the SCBS group compared to the DSBCS group. The difference between the two groups is statistically significant (p <0.001). Level of aggrecan obtained in the SBCS group was 30.71 ± 4.50 ng/ml compared with DSBCS group which was 8.41 ± 2.38 ng/ml. The mean level of aggrecan is higher in the SBCS group and the difference between two groups is statistically significant (p<0.001).

Transforming Growth Factor-*β* level of SCBS group was 130.12 ± 7.73 ng/ml and DSCBS group was 16.39 ± 4.49 ng/ml. The mean level of TGF-*β* was higher in the SBCS group compared to the DSBCS group and the difference is statistically significant (p <0.001).  Table 3 showed biochemical characteristic result in both groups.

### 3.5. Histological Examination

**Figure 9:**
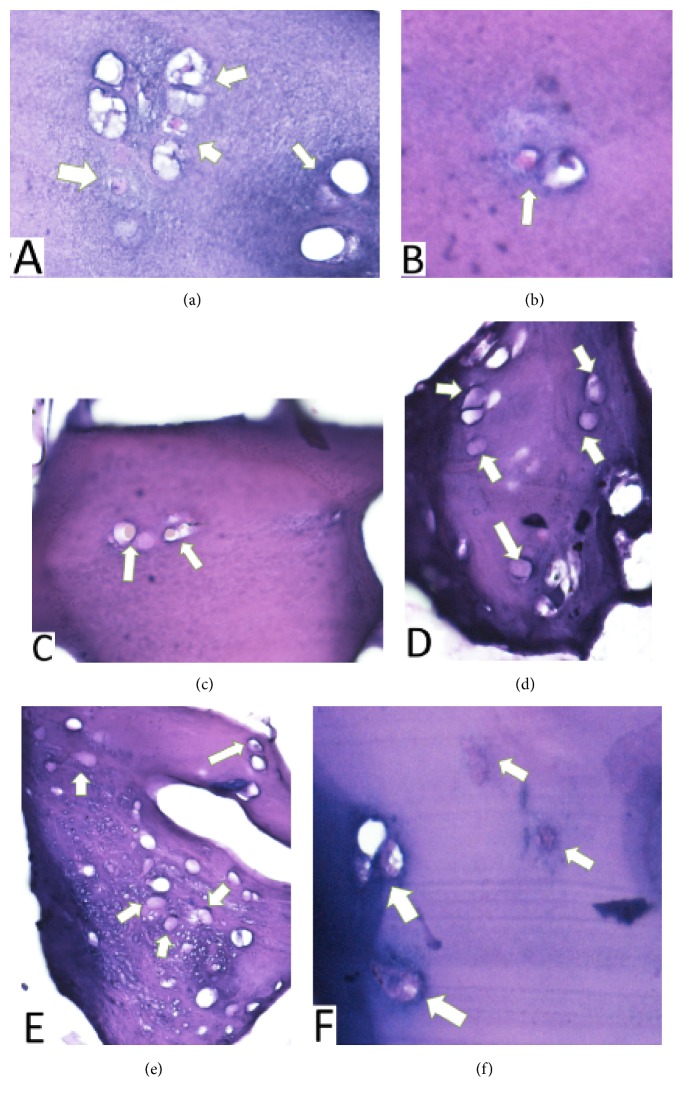
Hematoxylin-Eosin staining in 2 weeks after hBM-MSCs seeding. (a-c) SBCS group and (d-f) DSBCS group. White arrow showed chondrocytes.

**Figure 10:**
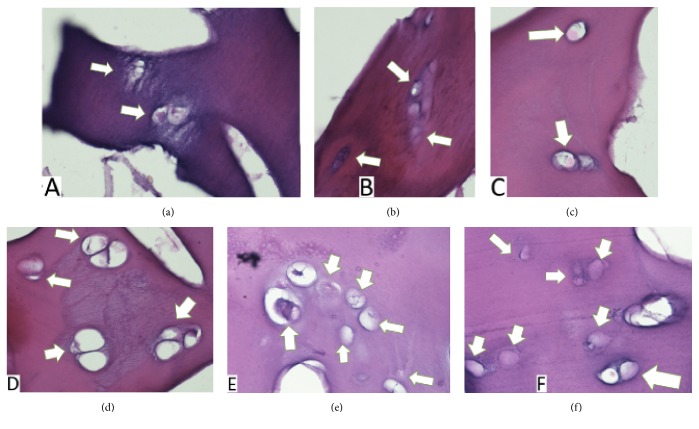
Hematoxylin-Eosin staining in 4 weeks after hBM-MSCs seeding. (a-c) SBCS group and (d-f) DSBCS group. White arrow showed chondrocytes.

Hematoxylin-eosin staining was carried out in weeks 2 and 4 after seeding of hBM-MSCs in vitro. Chondrocytes were found within the sponge cartilage bovine scaffold and can be seen in Figures 9 and 10.

Table 4 showed that chondrocytes count in weeks 2 and 4 was higher in DSBCS group compared to SBCS group, but there was not statistically significant difference between the two groups (p=0.469, p=0.215).

## 4. Discussion

The development of tissue engineering, with scaffold as one of the necessary element, plays an important and promising role in articular cartilage regeneration. Ideal scaffold in cartilage tissue engineering includes high porosity and pore interconnectivity [13]. We found that the mean porosity in SBCS group and DSBCS group was 90.07 ± 4.64% and 88.93 ± 4.18%, respectively. There was no statistical difference between the two groups (p=0.473). High porosity, usually between 80-90%, is enough to help in vitro cell adhesion. Porosity improves circulation of nutrients and oxygen to help the cells proliferate [14, 15].

The mean diameters of SBCS group and DSBCS group were 111.83 ± 14.23 *μ*m and 105.29 ± 11.14 *μ*m, respectively. The pore diameter of SBCS group was greater, but not statistically significant (p=0.142). Cell function and new tissue regeneration both depend on the porous size. The porous size must be in a range that facilitates cell penetration and migration during cell hatching, nutrient diffusion, and removal of metabolic substances and provides a three-dimensional environment capable of inducing cell coupling and differentiation [16]. Pore interconnectivity was proven in both scaffold groups. The interconnected pore of porous scaffold made of collagen facilitates the smooth distribution of a number of cells throughout the scaffold. Therefore, cells can be transmitted smoothly and distributed homogeneously not only in the larger porous regions but also in smaller pore areas [17].

Song et al., in their study, reported that large porous diameter between 100-300 *μ*m has an environment conducive to the adhesion and proliferation of cells [18]. Pore diameter of 100-150 *μ*m scaffold design is applicable for articular cartilage engineering [19]. In this study, SBCS and DSBCS groups had a mean diameter similar to the porous which is 105-111 *μ*m and this range is included in the criteria of a good size porous diameter as the cartilage scaffold according to Janik et al. If the size of the porous diameter is too small, the risk of porous occlusion due to higher cells can result in the circulation of metabolic substances being inhibited, affecting cell viability [20].

Both groups of sponge bovine cartilage scaffold have good water absorption and mechanical characteristics in tissue engineering [20]. The result showed that chondrocytes were found in both cell-seeded SBCS and DSBCS groups. Sponge bovine cartilage scaffold supports cell adhesion, transport of nutrients and residual metabolism, proliferation and chondrogenic differentiation of stem cells.

Evidence of pore interconnectivity, high porosity, water uptake properties, and ideal diameter distribution in both scaffold groups made the sponge bovine cartilage scaffold become a promising choice. Strongly supported by the ability to facilitate differentiation of hBM-MSCs into chondrocytes, this study found chondrocytes on histology examination of hematoxylin-eosin in the second and fourth weeks.

The porous diameter of SBCS and DSBCS groups ranged from 34.8-372.5 *μ*m and 14.7–332.7 *μ*m, respectively. These sizes belong to acceptable characteristic of pore diameter to facilitate good cell adhesion and water absorption. Therefore, our biodegradable porous sponge cartilage bovine scaffold would optimize cell seeding and filling.

Type II collagen-based scaffold is able to reproduce natural collagen to form extracellular matrix under optimal conditions [21]. Collagen-based scaffolds show the ability to form the same new cartilage structure as normal cartilage structures. Collagen-based scaffolds have low immunogenicity, porous structure, good biocompatibility, and biodegradability but have a disadvantage of minimal mechanical resistance. Collagen scaffold deficiency can be anticipated by combining with natural or synthetic polymer scaffolds [22].

Aggrecan has the ability to bind to hyaluronan and chondrocytes, stabilizing aggrecan fixation on the extracellular matrix to form cross-link network bonds. Aggrecan is a component of the pericellular matrix that maintains matrix homeostasis [23]. Some attempts to increase the strength of natural scaffolds, such as collagen, are by adding aggrecan in the manufacturing process [24]. The results showed that SBCS group had a higher content of aggrecan than DSBCS group. The high content of aggrecan and collagen type 2, especially in the SBCS group, is a good combination in terms of increasing the mechanical strength of the scaffold. It is expected that the sponge bovine cartilage scaffold studied is able to withstand normal joint loads, especially the knee so that the function as a scaffold can be maximized.

Aggrecan has in vitro chondrogenic properties [25]. Thus, the sponge bovine cartilage scaffold can trigger stem cells differentiation. It is supported by the results of our study that hematoxylin-eosin showed chondrocytes within scaffold at the second and fourth weeks after hBM-MSCs seeding.

Transforming Growth Factor-*β* levels were much higher in the SBCS group than in the DSBCS group. The difference in the mean number of TGF-*β* is statistically significant (p<0.001). This data shows that the decellularization process can reduce TGF-*β* levels. TGF-*β* has been used widely to induce chondrogenesis in mesenchymal stem cells, increase extracellular matrix production, and trigger the expression of type II collagen and aggrecan. A level of 10 ng/ml is most commonly used to induce chondrogenesis [26]. Exposure to TGF-*β* in the first week is very important for triggering chondrogenesis of mesenchymal stem cell [27]. Levels of TGF-*β* average of 130.12 ± 7.73 ng/ml in the SBCS group is expected to induce chondrogenesis of hBM-MSCs on scaffold without added external growth factors. Level of TGF-*β* 16.39 ± 4.49 ng/ml in the DSBCS group was also able to facilitate the differentiation of chondrocytes and can be seen through hematoxylin-eosin staining in 2 and 4 weeks. SBCS and DSBCS groups contain statistically different levels of TGF-*β* but both can facilitate the chondrogenic differentiation of hBM-MSCs.

Our sponge bovine cartilage scaffold contained an extracellular matrix component which consists of type II collagen and aggrecan, and growth factors TGF-*β*. SBCS group contained higher levels of aggrecan, type II collagen, and TGF-*β* than DSBCS group, indicating that sponge bovine cartilage scaffold can be called an ECM (extracellular matrix) based scaffold. Despite functioning as a wake-up frame, sponge bovine cartilage scaffolds can provide necessary growth factors as biochemical signals for cell proliferation and differentiation without adding external growth factors.

Ability to absorb water from sponge bovine cartilage scaffold was measured by swelling ratio. Water absorption, which is defined as the ability of a scaffold to maintain water and water permeation, has become one of the important requirements for biomedical scaffolds [28]. This has proven to be an important factor for the absorption of body fluids and for the transfer of oxygen, nutrients, and metabolites in the scaffold so that it is useful during encapsulation. The ability of scaffold to absorb water has an effect on cell proliferation and structural morphology in tissue regeneration [29]. Collagen-based porous scaffolds show significantly higher water content than synthetic biopolymers. Water absorption by collagen scaffold is ten times greater than the original weight, probably due to its hydrophilic nature and the porous structure of scaffold [30].

This study documented swelling ratio in both groups. The SBCS group absorbed water higher than DSBCS group (594.66 ± 5.08% vs. 387.50 ± 2.35%) in first 15 minutes. Water absorption in the first minutes was high and started to stabilize after the 30 minutes. This result showed that both SBCS and DSBCS groups have hydrophilic properties, which are very attractive for water. This hydrophilic nature adds an advantage of facilitating the entry of nutrients into the scaffold, thus providing an opportunity for better growth of chondrocytes.

Rate of biodegradation should be in accordance with the rate of tissue regeneration. A good scaffold can survive as a scaffold for colonization, proliferation, and cell differentiation but must be completely degraded after the regeneration process ends [31]. Scaffold biodegradation depends on the internal content, hydrophilic properties, and the ability to absorb water. We recommended that the scaffold begin to degrade when the tissue begins to regenerate and preferably when the scaffold is degraded, and even though the scaffold mass is reduced, the porous quality must be maintained well.

The DSBCS group had a higher average degradation rate of 16.04 ± 0.13%, while SBCS group was 12.56 ± 0.41% in two weeks. This result reflects the biodegradable nature of the scaffold. The SBCS group was slower to be degraded perhaps because it contains more extracellular matrix levels than the DSBCS group.

In a study conducted by Sutherland et al., bone marrow mesenchymal stem cells cultured into decellularized cartilage scaffolds express a chondrogenic marker [32]. However, in cartilage scaffold without decellularization, proteoglycan retention such as aggrecan is beneficial because of its conductive properties [33]. Decellularization is an effort to minimize the risk of stem cell infection by scaffold. The decellularization process, both physically and chemically, reduces the biochemical content but does not damage the structure, inhibit, or reduce stem cell differentiation within scaffold [34]. This study showed that hBM-MSCs differentiated into chondrocytes evidenced on histological examination at second and fourth weeks in both SBCS and DSBCS groups.

We realized that the shortcomings in our study are using simple porosity measurement methods. We hope further researches will utilize a more meticulous method in porosity measurement.

## 5. Conclusion

Based on the results, it can be concluded that both sponge bovine cartilage scaffolds, either with decellularization (DSBCS) or without decellularization (SBCS), have fulfilled the necessary requirement of physical, biochemical, biodegradability, and water absorption characteristics as an ideal biodegradable porous sponge bovine cartilage scaffold. These characteristics induced and facilitated the cell proliferation and differentiation of human bone marrow mesenchymal stem cells (hBM-MSCs) in vitro. However, further research is still needed so that porous biodegradable sponge Bovine cartilage scaffold preparations can be applied clinically. As for future work, we will conduct an in vivo experimental research based on this study.

## Figures and Tables

**Table 1:** Pore diameter distribution within SBCS group and DSBCS group.

**Pore Diameter Range**	**SBCS group**	**DSBCS group**
< 50 *μ*m	9.40%	19.30%
50 – 100 *μ*m	37.10%	33.80%
100 – 150 *μ*m	38.20%	34.50%
150 – 200 *μ*m	9.20%	7.20%
200 – 250 *μ*m	3.80%	3.60%
> 250 *μ*m	2.30%	1.60%

**Table 2:** Average pore diameter and porosity in SBCS group and DSBCS group.

**Physical Characteristics**	**SBCS Group **	**DSBCS Group **	**p value**
	**(n=16)**	**(n=16)**	
Porosity (mean ± s.d) (%)	90.07 ± 4.64	88.93 ± 4.18	0.473
Average pore diameter (mean ± s.d) (*μ*m)	111.83 ± 14.23	105.29 ± 11.14	0.142

**Table 3:** Biochemical characteristics result in SBCS group and DSBCS group.

**Biochemical Characteristics**	**SBCS Group **	**DSBCS Group **	**p value**
	**(n=16)**	**(n=16)**	
Type II Collagen (mean ± s.d)ng/ml	380.78 ± 18.63	64.83 ± 13.54	0.001
Aggrecan (mean ± s.d)ng/ml	30.71 ± 4.50	8.41 ± 2.38	0.001
TGF-*β* (mean ± s.d)ng/ml	130.12 ± 7.73	16.39 ± 4.49	0.001

**Table 4:** Chondrocytes count in SBCS group and DSBCS group.

**Time of Evaluation**	**Chondrocytes count (mean ± s.d)**		**p value**
	**SBCS group (n=3)**	**DSBCS group (n=3)**	
2 weeks	7.33 ± 1.52	10.00 ± 5.57	0.469
4 weeks	16.00 ± 3.0	21.33 ± 5.51	0.215

## Data Availability

The data used to support the findings of this study are included within the article.
